# Key concepts in consumer and community engagement: a scoping meta-review

**DOI:** 10.1186/1472-6963-14-250

**Published:** 2014-06-13

**Authors:** Pooria Sarrami-Foroushani, Joanne Travaglia, Deborah Debono, Jeffrey Braithwaite

**Affiliations:** 1 Centre for Clinical Governance Research, Australian Institute of Health Innovation (AIHI), University of New South Wales (UNSW), Sydney, NSW 2052, Australia; 2 School of Public Health and Community Medicine (SPHCM), University of New South Wales (UNSW), Sydney, NSW 2052, Australia

**Keywords:** Consumer and community engagement (CCE), Concepts, Shared decision making (SDM), Self-management, Peer support

## Abstract

**Background:**

Although consumer and community engagement (CCE) in health care is receiving increasing attention, research and practice in this area are hampered by the variability of concepts and terminology commonly employed. This scoping meta-review aims to identify key CCE concepts and examine terminology used to describe them.

**Methods:**

In a scoping meta-review, an extensive list of 47 phrases and 11 Medical Subject Headings (MeSH) was used to undertake a comprehensive and systematic search in PubMed Central, Embase, EBM reviews, CINAHL, APAPsycNET, and Scopus.

**Results:**

59 systematic reviews met the selection criteria and were included in the final analysis. The analysis identified nine different concepts related to CCE: shared decision making, self-management, CCE in health care systems, community-based health promotion, providing access to health care, rehabilitation, participation in research, collaboration in research design and conduct, and peer support. The identified concepts differ from each other in many aspects including the aim of the activity, the role of consumers and the type of professionals’ involvement. Each concept was described by a range of terms, with some terms shared by different concepts. In addition, two overlapping concepts of patient-centeredness and patient empowerment were recognised.

**Conclusions:**

This study describes CCE-related key concepts and provides new insight into their relationship with different CCE-related terms. Identification of key CCE-related concepts and terms will be useful to focus future studies and initiatives and enhance production of CCE-related evidence.

## Background

Policy-makers, researchers and the public have paid growing attention to the role of consumer and community engagement (CCE) in health care over the last decade [1]. Yet there is no single, agreed definition of CCE. CCE can occur in different fields and for a variety of purposes. The relationship between consumers and community members with health care systems is diverse. Szasz and Hollender [2] identify three roles that patients might adopt in their relationship with clinicians: recipients, co-operators, and active participants. Bowen et al. [3] have suggested a ‘continuum of community engagement’ in which engagement of the community has three potential stages in which the community adopts: a passive role primarily receiving information; a more active but still predominantly recipient role; and shared decision making role with more or less equal positioning.

In addition, it seems that a set of overlapping terms is used to signify all or parts of the same core concept of CCE. This exemplifies the arbitrary relationship between words and underlying concepts suggested by De Saussure [4]. CCE is referred to using terms including ‘shared decision making (SDM)’, ‘patient participation’, and ‘community engagement’, amongst numerous others.

Several authors have identified that the use of mutual terminology for disparate activities can lead to controversy [5-9]. This is one of the reasons why providing an evidence base for the efficacy of CCE has remained a challenge. While the principle of CCE is gaining momentum in health systems across the world, evidence for its effectiveness in improving patient outcomes and its cost effectiveness remains relatively weak [1,10-13]. This scoping meta-review aims to identify different CCE-related concepts and examine the terminology used to describe those concepts.

## Methods

This study is based on an innovative method, scoping meta-review, which combines scoping review and meta-review methods. A scoping review is an emerging literature review methodology that is useful to map out a field of interest [14,15]. This method can be used as a transparent technique to map the literature and address broad research questions on a topic [14]. Meta-reviews refer to activities which synthesise evidence from an overview of systematic reviews [16]. We used scoping review methodology to overview CCE-related systematic reviews. The appropriateness of this method was identified based on a non-systematic preliminary review that indicated that the field is diverse and complex. Therefore we needed a scoping review methodology to map CCE-related concepts. In addition, based on the preliminary review, we noted that there were many systematic reviews on various aspects of CCE. Therefore, it was feasible to undertake a scoping overview of existing systematic reviews on CCE in health care. The advantage of relying on systematic reviews was the possibility of presenting a robust and reliable picture of the field. Each paper included using this method is a systematic review that has appraised a number of studies. Furthermore, systematic reviews present their search terms, which is particularly relevant to the aim of a study exploring various CCE-related phrases.

In conducting the preliminary review, we identified a comprehensive list of nine medical subject headings (MeSH) and 47 phrases search terms (see Additional file 1). The extensive list was used to ensure our sample would include various types of CCE-related concepts.

We searched six major databases in health care and medicine: PubMed Central; Embase, EBM reviews; CINAHL; APAPsycNET; and Scopus. Systematic reviews examining CCE in health care were included, incorporating all health care clients irrespective of the health care problem. Citations were downloaded into EndNote X5, a bibliographic database. This software assisted management of our review, including identification of duplications, browsing titles and abstracts, and saving the review results.

**Figure 1:**
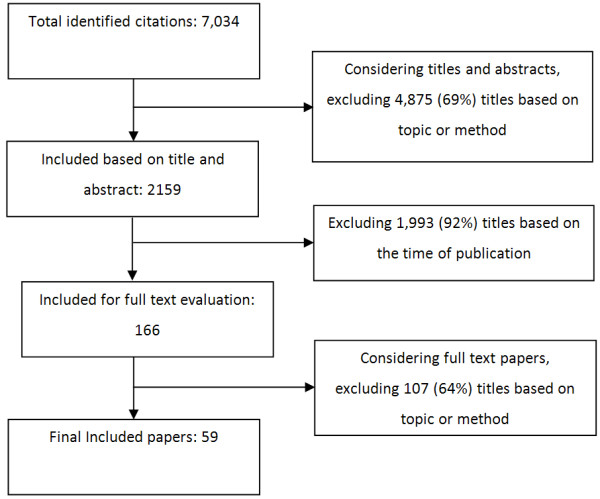
**Relationship between CCE-related concepts and related phrases.** Concepts are labelled in circles with bold font, which are connected to the related phrases illustrated by squares. Phrases are labelled only if they are shared by three or more concepts. This Figure is illustrating the overlaps between phrases for different concepts, and also it illustrates that each concept is described by different phrases.

The search excluded: studies presenting data from a single study, opinions, books, chapters, discussions, and letters and publications in languages other than English. No geographical restrictions were placed on the citations. Initially no time limit was considered. However, following the identification of 2,159 citations for interrogation, the search strategy was revised in order to obtain a more manageable sample. The revised search strategy included only those systematic reviews published between January 2010 and October 2011. Revising the search strategy is an acceptable step in a scoping review [14] and as each systematic review examines published studies from previous years, we were able to indirectly access studies published prior to 2010. The first author screened the abstracts against the selection criteria. Irrelevant references were excluded on the basis of the relevance of their title and abstract. The full text was obtained for the remaining references and evaluated against the selection criteria by the first and second author. Papers were excluded if they were not related to CCE or if they were not systematic reviews. An appraisal tool developed by the Public Health Resource Unit, England was consulted to decide whether papers fulfilled the criteria of a systematic review [17]. Data analysis involved several steps. Following a close reading of the selected papers, each paper was allocated to an emerging category of CCE-related concepts. After identifying all emergent categories of CCE-related concepts and allocating all of the selected papers to a category, phrases that were used to describe each concept were identified. At this point, a table of CCE-related concepts was created. We used Cytoscape software, to produce a visual representation of the complex relationship between phrases and concepts (Figure 1).

## Results

**Figure 2:**
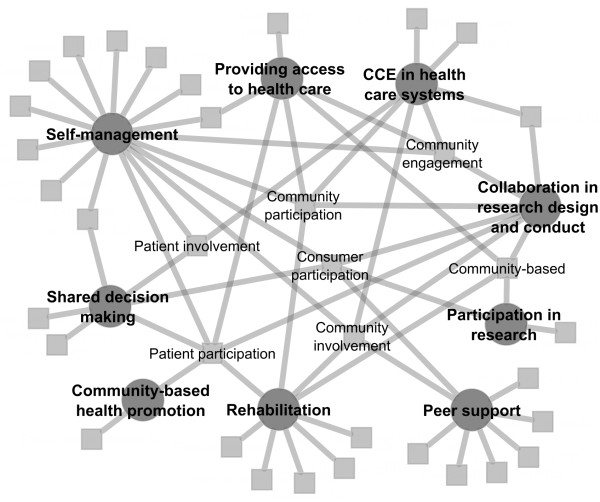
Summary of study selection and exclusion.

The results of the scoping meta-review are illustrated in Figure 2. Initially our search across the academic literature data bases identified 10,078 references. Following removal of 3,044 duplicates, 7,034 references remained. These references were evaluated according to their titles and abstracts and 4,875 papers were excluded based on their topic or method. In the next stage, 1,993 papers published before 2010 were excluded. There were 166 papers included for full text review. During the final stage, 107 papers were excluded based on topic relevance or methodology. There were 59 papers remaining that were analysed and evaluated.

### Emerging concepts

Based on the analysis of the included systematic reviews (n = 59), nine different categories of concepts emerged (Table 1). These concepts refer to different types of CCE-related activities, processes or mechanisms; they involve different types of health care professionals and have different aims; and they also present different types of roles for consumers and community members (Table 1).

**Table 1:** A summary of nine different CCE-related concepts that emerged through analysis of literature

**Identified concepts related to CCE**	**Phrases used to describe each concept**	**Aim**	**Role of consumers and/or community members**	**Involvement of professionals**
Shared decision making	Consumer involvement	Improving own health	Active participation in the process of decision making for their own conditions	Activity is a joint process involving health care providers
	Consumer participation			
	Patient involvement			
	Patient participation			
	Patient preference			
	Shared decision making			
Self-management: Participation of patients in their own medical treatment	Community-based intervention	Improving own health	Co-operating and active participation in their own health management	Activity is an action by consumers or community members
	Community engagement			
	Community involvement			
	Community participation			
	Consumer engagement			
	Consumer involvement			
	Consumer participation			
	Patient activation			
	Patient-centred (care)			
	Patient-driven			
	Patient education			
	Patient engagement			
	Patient involvement			
	Patient participation			
	Self-care			
	Self-management			
CCE in health care systems	Community engagement	Improving health in general	Active participation in health care systems	Activity is a joint process involving employees of health care systems
	Community involvement			
	Community participation			
	Community wide interventions			
	Decision-making			
	Patient involvement			
	Public involvement			
Community-based health promotion	Patient participation	Either improving own health or improving health in general	Active participation in actions leading to health promotion	Activity is an action by consumers or community members
	Public engagement			
Providing access to health care	Community-based (screening)	Improving own health	Recipient of health care	Activity is a joint process involving health care providers
	Community demand			
	Community engagement			
	Community participation			
	Patient engagement			
	Patient participation			
Rehabilitation: increasing social participation	Community-based rehabilitation	Improving own health	Active participation in social life	Activity is an action by consumers or community members
	Community integration			
	Community involvement			
	Community participation			
	Person-centred (planning)			
	Patient participation			
	Social involvement			
	Social engagement			
	Social participation			
Participation in research	Community-based (research)	Improving health in general	‘Recipient’ of research	Activity is a joint process involving researchers
	Consumer participation			
	Patient recruitment			
Collaboration in research design and conduct	Community-based (research)	Improving health in general	Active participation in the research design and conduct	Activity is a joint process involving researchers
	Community engagement			
	Community involvement			
	Community participation			
	Consumer participation			
	Patient participation			
	Public involvement			
Peer support: patients as health care providers	Community involvement	Improving health in general	Active participation in the care of other patients	Activity is an action by consumers or community members
	Consumer-led services			
	Consumer participation			
	Consumer provider			
	Empowerment			
	Peer-led (interventions)			
	Peer services			
	Peer support			

#### Shared decision making

Shared Decision Making (SDM) is hypothesised to promote active consumer involvement in health-related decisions [18]. As a concept it refers to a style of communication between a patient and clinician which aims to place patients’ preferences and values on a level comparable, although not the same as, clinical information [18].

The aim of SDM is to achieve positive outcomes for patients’ communication between the clinician and patient and helping patients to select better treatment options [18]. However, systematic reviews included in this study identified that evidence for SDM is mixed [19] and that SDM faces a number of challenges. For example, patients’ actual participation in SDM might be less than they prefer [20-22] and health care professionals may need guidance on how to implement SDM [22]. One systematic review reported that SDM had no effect on clinical outcomes in mental health [23].

#### Self-management

Self-management is undertaken by educating, supporting and encouraging patients to adopt an active role in their own health management [24-26]. Self-management is particularly relevant to patients affected by chronic diseases such as diabetes [27].

#### CCE in health care systems

CCE in health care systems refers to involvement of citizens in the design of health care policies, service delivery or interventions. This may include, for example, the design and introduction of new technologies in health care [28,29]. Both patients and members of the general public can be involved in health care systems. However, Menon et al. have noted that individual patients’ opinions about an approach may differ to those of the general public and it is therefore necessary to distinguish between the participant groups and to be transparent about whether the opinion of public or patients has been obtained [28].

#### Community-based health promotion

Community-based health promotions refer to the communities’ participation in actions and activities that improve the health of, and reduce risks to, those communities. Community members might, for example, be encouraged to participate in more physical activities to improve their wellbeing [30,31] or to participate in notification and control of infections [32,33]. More information is needed to support the efficacy of current approaches in increasing community participation [30].

#### Providing access to health care

We identified some phrases such as ‘community engagement’ or ‘patient participation’ that are used to describe provision of access to health care. Related systematic reviews described strategies employed to facilitate access to specific health care services, or the strategies employed to increase the use of health services by particular groups or individuals. For example, telemedicine has been used as a mechanism to increase the public’s access to health care [34]; or community reinforcement and family training have been used to engage treatment-resistant patients with substance abuse problems [35]. In contrast with SDM, the main intention of ‘providing access to health care’ is to provide health care, not necessarily an active involvement in decision making and health care.

#### Rehabilitation: increasing social participation

We identified that phrases such as ‘patient participation’ are used to describe rehabilitation of patients with limited mobility, such as stroke patients. The aim of ‘patient participation’ in this context is to increase patients’ participation in social activities [7,36] and participation is an index of patients’ health and rehabilitation [6]. It is worth noting that systematic reviews included in this study referred to difficulties in the definition and conceptualisation of ‘participation’ [6-8].

#### Participation in research

Phrases such as ‘consumer participation’ might seem to denote active involvement of patients in research. However, we identified that in some systematic reviews, ‘participation in research’ referred to recruitment of consumers and community members into research projects as passive participants and subjects of a study. These systematic reviews explored studies that aimed to enhance recruitment and retention of people with a specific health condition, age, or from a particular ethnic group into clinical research [37-40].

#### Collaboration in research design and conduct

In contrast to the previous concept, consumers and community members may be invited to actively collaborate in the design and conduct of research [41,42]. Within this domain, members of the public and patients are engaged with the research process in a variety of ways ranging from participation in ethics committees, through to roles on community advisory boards and to undertaking an active role as a co-investigators [43]. Members of the public actively contributing to research may be involved in: defining the scope of the study; recommending, identifying and evaluating the relevant literature; interpreting the findings; checking the consent processes and information sheets; examining the data collection processes; writing up the results; and reviewing outcome recommendations [44,45].

#### Peer support: patients as health care providers

Peer support refers to patients undertaking an active role in educating or providing care and treatment for other patients [46-49]. According to the included systematic reviews, this approach has been shown to be a successful mechanism for patient education and can contribute to changes in consumer and community members’ knowledge and norms [47,50].

Several challenges for this approach have been identified. More research is needed on consumer-led services [51]. Consumers providing peer support require training, supervision, and management [52]. More funding would be required to meet these challenges [46].

### Different phrases related to each concept

The first stage of the analysis included identifying different CCE-related concepts. In the next stage of the analysis, we explored phrases that were used to describe those concepts. We identified that each concept is described with a range of different phrases (Table 1).

### Different concepts related to each phrase

We undertook a network analysis of the concepts and related phrases and identified that despite the differences between various aspects of the concepts, similar phrases are used to describe them. For example, as illustrated in Figure 1, systematic reviews included in this study used ‘community engagement’ to describe ‘self-management’, ‘providing access to health care’, ‘CCE in health care systems’ and ‘collaboration in research design and conduct’.

### Other concepts that overlap with CCE

As expected the concepts that emerged in our study include activities, processes, and mechanisms that were described by one of our search terms (e.g. consumer engagement or patient participation). Our analysis also identified two additional concepts, patient-centeredness and patient-empowerment, that are not described by any of the search terms we used. Significantly, these concepts are highly related to and overlap with the concept of CCE in the systematic reviews analysed in this study.

Patient-centeredness or patient-centred care implies putting the patients’ needs and experiences as the first priority and at the heart of care. This includes considering patients’ psychological needs and engaging with patients’ life experience [53,54]. Patient-centeredness can incorporate the encouragement of patients’ participation in their health care and related decisions, as discussed in SDM [55]. However, patient-centeredness is not equal to SDM and is a broader approach focusing on the clinicians’ and services’ relationship with and stance towards patients, as well as the patients’ involvement in services.

Patient empowerment implies improving patients’ knowledge, control of and impact on their own health care [56,57]. Empowerment is defined in a variety of ways, such as supporting patient autonomy, and choice [58]. Therefore, this concept is closely related to other CCE-related concepts such as SDM, self-management and CCE in health care systems.

## Discussion

CCE is not defined uniformly and it does not incorporate a single concept and type of activity. This scoping meta-review identifies nine different CCE-related concepts that are described by assorted terms. Definitions of CCE may incorporate differing combinations of these concepts. That is, the relevance of some of the concepts will be determined by how CCE is defined. For example, this study identified ‘rehabilitation’ as a concept related to CCE, because it was described by phrases such as ‘patient participation’ (here ‘patient participation’ was used to describe participation of patients in normal social activities). However, if CCE is defined as the active involvement of patients and community members in health care, this definition will not necessarily involve participation of patients in social activities. The use of the same CCE-related terms to describe different concepts can lead to confusion and miscommunication. Identification of these various concepts described in this paper will be helpful for practitioners, researchers and policy-makers. They can be transparent on the range of concepts that are included in the definition of CCE they are adopting or creating.

It is notable that the identified concepts can be seen to be related to a particular group of health care professionals. For clinicians and health providers, *SDM, self-management, rehabilitation* and ‘*peer support’* are the relevant concepts. Those who work in health care organisations, such as health policy-makers, might wish to advocate all concepts, but ‘*CCE in health care systems’*, ‘*community-based health promotion’*, and ‘*providing access to health care’* are core to their direct responsibilities. Finally, ‘*participation in research’* and ‘*collaboration in research design and conduct’* are more squarely related to researchers and academics. This is not, of course, an exclusive categorisation. Any practitioner or policy-maker might deal with any concept introduced in this paper. But we suggest that having this map may assist interested parties to be fully aware of the range of possibilities and types of CCE-related activities and mechanisms.

Although the identified concepts vary considerably in their aims (i.e. improving one’s own health versus improving health in general), participants (e.g. clinicians, researchers or policy-makers) and locations (e.g. health care centres, universities, and health organisations), the majority of these concepts are directed at expanding the role(s) consumers and community members undertake in health care (Table 1). As such they redress a more historical approach where patients are expected to be recipients of health care, rather than being active players within it [59]. CCE can therefore be viewed as presenting a change in historical focus of health care and a challenge in health care settings [53].

Other authors have indicated that concepts and terms underpinning CCE such as engagement, participation, empowerment, and involvement are interpreted differently and are not defined alike [22,41,60]. This may explain the finding of this study that some phrases like ‘community engagement’ are shared by different concepts and as such become umbrella terms [29,60].

Despite the large volume of evidence that exists in relation to CCE, according to the included systematic reviews, there is a shortage of high quality evidence to support CCE-related concepts. We propose that as long as these activities are referred to with wide, umbrella phrases, this uncertainty and complexity will continue. Supporting evidence for ‘community engagement’, for example, is meaningless unless the intended concept is specified.

In addition, it might be interesting to debate over various phrases used and to discuss which is the most relevant phrase to utilise. A consensus-building study or process can be attempted to achieve agreement over a universally used technical term. The framework of nine different concepts and their related phrases may be helpful in this process. However, while this has not yet been achieved, it will be crucial over time to be transparent on the concepts intended by chosen phrases. For example, one may select the phrase of ‘patient participation’, and be transparent by indicating that this includes only SDM and self-management.

Previous attempts to overcome complexities in this field involved classifying activities. Bowen et al. [3], for example, introduce a ‘continuum of community engagement’ and divided engagement strategies into three categories: ‘transactional, transitional, and transformational engagement’. Another framework provided by Mittler et al. [12] emphasises differentiation of engaged behaviours and activation (the motivation for engaged behaviours). The authors of the current study have also introduced a model that identifies eight key elements for planning for CCE. These elements are: identification of aims; type of activity; participants; preparedness; method of engagement; measurement method; barriers; and facilitators [61]. The scoping meta-review identifies and details types of CCE-related concepts thereby facilitating the application of CCE models.

### Limitations

We have selected and explored a sample of academic papers, which did not directly include non-systematic reviews and grey literature. In addition, despite our efforts to include a wide range of papers by utilising an extensive list of 47 search phrases, there might be other CCE-related concepts that are not identified in this paper. Finally, qualitative analysis of concepts and phrases is inevitably a subjective process. Future work should examine this framework and attempt further refinement of CCE-related concepts.

## Conclusions

This scoping meta-review provides new insights into the relationship of phrases and concepts related to the field of CCE in health care. By identifying the specific concepts related to CCE, this study can assist more focused evaluations of the current evidence, and more importantly, enhance the production of new evidence.

## Abbreviations

CCE: Consumer and community engagement; MeSH: Medical subject heading; SDM: Shared decision making.

## Competing interests

The authors declare that they have no competing interests.

## Authors’ contributions

PSF designed the search strategy, carried out the systemic search, and drafted the manuscript. JT participated in the design of the review and helped in undertaking the search and drafting the manuscript. DD supported the study and participated in drafting the manuscript. JB supervised progress and participated in the design of the study and drafting the manuscript. All authors read and approved the final manuscript.

## Authors’ information

PSF is a medical sociologist with a doctoral degree in medicine and a PhD in sociology. His broad expertise includes the study of social aspects of health and medicine for more than a decade. He has extensive international research expertise and has worked in Iran, the United Kingdom and Australia focusing on consumer and community engagement, clinical variation, and mental health. He is active in supervising postgraduate students undertaking research on consumer and community engagement in health care at UNSW.

JT has been involved in health services education and research for over 20 years, actively inquiring into and promoting and developing the fields of diversity, patient safety and inter-professional learning and practice across the Australian and Italian health systems. She has led research and evaluation projects on a range of topics relating to patient safety, the quality and equity of service provision in aged and health care, communication, collaboration, peer support, diversity, ethnicity, cultural competence, disability and inter-professionalism. Dr Travaglia worked most recently in the Centre on an evaluation of inter-professional practice and learning across an entire health system, and currently works on projects concerning consumer engagement, vulnerability, patient safety, and comparative international health systems studies.

DD is a research officer in the Centre for Clinical Governance Research. She is a registered nurse and midwife with experience in both rural and metropolitan acute care settings. Ms Debono graduated with a Bachelor of Arts degree majoring in Psychology and Sociology. Her Honours Thesis investigated automatic and controlled cognitive processing in the elderly and her research interests are medication error, patient safety and workarounds. Ms Debono is conducting projects and providing research support in a range of areas and is undertaking a PhD focusing on workarounds in health care.

JB is a leading health services researcher known internationally for his work investigating the culture and structure of acute settings, leadership, management and change in health sector organisations, quality and safety in health care, accreditation and surveying processes in an international context and the restructuring of health services. Professor Braithwaite has published extensively (more than 300 refereed contributions, and 500 total publications) about organisational, social and team approaches to care which has raised the importance of these both here and internationally. Theories and ideas he has helped shape, formulate or devise, and provided research findings for, are now in common use: multi-method, triangulated approaches to research, the boundary-less hospital, accreditation models in general practice and beyond, clinician-managers as key players in reform initiatives, fundamental principles for the governance of health systems, diversity in clinical professional groups, and inter-professional learning and culture change rather than restructuring as a more sustainable strategy for reform.

## Pre-publication history

The pre-publication history for this paper can be accessed here:

http://www.biomedcentral.com/1472-6963/14/250/prepub

## Supplementary Material

Additional file 1 Search strategy. Click here for file

## References

[B1] Crawford MJ Rutter D Manley C Weaver T Bhui K Fulop N Tyrer P Systematic review of involving patients in the planning and development of health care BMJ 2002 14 7375 1263 12458240 10.1136/bmj.325.7375.1263 PMC136920

[B2] Szasz TS Hollender MH A contribution to the philosophy of medicine: the basic models of the doctor-patient relationship Arch Intern Med 1956 14 5 585 10.1001/archinte.1956.00250230079008 13312700

[B3] Bowen F Newenham-Kahindi A Herremans I When suits meet roots: the antecedents and consequences of community engagement strategy J Bus Ethics 2010 14 2 297 318

[B4] De Saussure F Nature of The Linguistic Sign Course in General Linguistics 1916

[B5] Chung EY-H Packer T Yau M When East meets Wests: community-based rehabilitation in Chinese communities Disabil Rehabil 2011 14 8 697 705 20690860 10.3109/09638288.2010.506240

[B6] Eyssen IC Steultjens MP Dekker J Terwee CB A systematic review of instruments assessing participation: challenges in defining participation Arch Phys Med Rehabil 2011 14 6 983 997 21621675 10.1016/j.apmr.2011.01.006

[B7] Levasseur M Richard L Gauvin L Raymond E Inventory and analysis of definitions of social participation found in the aging literature: proposed taxonomy of social activities Soc Sci Med 2010 14 12 2141 2149 21044812 10.1016/j.socscimed.2010.09.041 PMC3597625

[B8] Cook F Oliver C A review of defining and measuring sociability in children with intellectual disabilities Res Dev Disabil 2011 14 1 11 24 21036013 10.1016/j.ridd.2010.09.021

[B9] Dijkers MP Issues in the conceptualization and measurement of participation: an overview Arch Phys Med Rehabil 2010 14 9 Suppl S5 S16 20801280 10.1016/j.apmr.2009.10.036

[B10] Simpson EL Barkham M Gilbody S House A Involving service users as researchers for the evaluation of adult statutory mental health services Cochrane Database Syst Rev 2009 14 1

[B11] Nilsen ES Myrhaug HT Johansen M Oliver S Oxman AD Methods of consumer involvement in developing healthcare policy and research, clinical practice guidelines and patient information material Cochrane Database Syst Rev 2006 14 CD004563 16856050 10.1002/14651858.CD004563.pub2 PMC6464810

[B12] Mittler JN Martsolf GR Telenko SJ Scanlon DP Making sense of “consumer engagement” initiatives to improve health and health care: a conceptual framework to guide policy and practice Milbank Q 2013 14 1 37 77 23488711 10.1111/milq.12002 PMC3607126

[B13] Hibbard JH Greene J What the evidence shows about patient activation: better health outcomes and care experiences; fewer data on costs Health Aff (Millwood) 2013 14 2 207 214 23381511 10.1377/hlthaff.2012.1061

[B14] Arksey H O’Malley L Scoping studies: towards a methodological framework Int J Soc Res Meth 2005 14 1 19 32

[B15] Levac D Colquhoun H O’Brien KK Scoping studies: advancing the methodology Implement Sci 2010 14 69 20854677 10.1186/1748-5908-5-69 PMC2954944

[B16] Higgins J Green S Cochrane Handbook for Systematic Reviews of Interventionsversion 5.1.0 [Updated March 2011] Available From 2011 The Cochrane Collaboration http://www.cochrane-handbook.org

[B17] The public health resource unit Critical Appraisal Skills Programme Tools Oxford available at http://www.casp-uk.net/#!casp-tools-checklists/c18f8 access date: 06.10.2011

[B18] Curtis LC Wells SM Penney DJ Ghose SS Mistler LA Mahone IH Delphin-Rittmon M del Vecchio P Lesko S Pushing the envelope: shared decision making in mental health Psychiatr Rehabil J 2010 14 1 14 22 20615840 10.2975/34.1.2010.14.22

[B19] Perestelo-Perez L Gonzalez-Lorenzo M Perez-Ramos J Rivero-Santana A Serrano-Aguilar P Patient involvement and shared decision-making in mental health care Curr Clin Pharmacol 2011 14 2 83 90 21592063 10.2174/157488411796151192

[B20] Tariman JD Berry DL Cochrane B Doorenbos A Schepp K Preferred and actual participation roles during health care decision making in persons with cancer: a systematic review Ann Oncol 2010 14 6 1145 1151 19940010 10.1093/annonc/mdp534 PMC4200024

[B21] Belanger E Rodriguez C Groleau D Shared decision-making in palliative care: a systematic mixed studies review using narrative synthesis Palliat Med 2011 14 3 242 261 21273220 10.1177/0269216310389348

[B22] Moore L Kirk S A literature review of children’s and young people’s participation in decisions relating to health care J Clin Nurs 2010 14 15–16 2215 2225 20659201 10.1111/j.1365-2702.2009.03161.x

[B23] Duncan E Best C Hagen S Shared decision making interventions for people with mental health conditions Cochrane Database Syst Rev 2010 14 1 29 10.1002/14651858.CD007297.pub2 PMC7209977 20091628

[B24] Schwappach DLB Wernli M Medication errors in chemotherapy: incidence, types and involvement of patients in prevention. A review of the literature Eur J Cancer Care (Engl) 2010 14 3 285 292 19708929 10.1111/j.1365-2354.2009.01127.x

[B25] Car J Lang B Colledge A Ung C Majeed A Interventions for enhancing consumers’ online health literacy Cochrane Database Syst Rev 2011 14 CD007092 21678364 10.1002/14651858.CD007092.pub2 PMC6464831

[B26] Ryan R Santesso N Hill S Lowe D Kaufman C Grimshaw J Consumer-oriented interventions for evidence-based prescribing and medicines use: an overview of systematic reviews Cochrane Database Syst Rev 2011 14 CD007768 21563160 10.1002/14651858.CD007768.pub2

[B27] Minet L Møller S Vach W Wagner L Henriksen JE Mediating the effect of self-care management intervention in type 2 diabetes: a meta-analysis of 47 randomised controlled trials Patient Educ Couns 2010 14 1 29 41 19906503 10.1016/j.pec.2009.09.033

[B28] Menon D Stafinski T Role of patient and public participation in health technology assessment and coverage decisions Expert Rev Pharmacoecon Outcomes Res 2011 14 1 75 89 21351860 10.1586/erp.10.82

[B29] Attree P French B Milton B Povall S Whitehead M Popay J The experience of community engagement for individuals: a rapid review of evidence Health Soc Care Community 2011 14 3 250 260 21138495 10.1111/j.1365-2524.2010.00976.x

[B30] Baker PR Francis DP Soares J Weightman AL Foster C Community wide interventions for increasing physical activity Cochrane Database Syst Rev 2011 14 6 436 437 10.1002/14651858.CD008366.pub2 21491409

[B31] Hartman MA Hosper K Stronks K Targeting physical activity and nutrition interventions towards mothers with young children: a review on components that contribute to attendance and effectiveness Public Health Nutr 2011 14 8 1364 1381 20633316 10.1017/S1368980010001941

[B32] Abad-Franch F Vega MC Rolon MS Santos WS Rojas de Arias A Community participation in Chagas disease vector surveillance: systematic review PLoS Negl Trop Dis 2011 14 6 e1207 21713022 10.1371/journal.pntd.0001207 PMC3119642

[B33] Atkinson JA Vallely A Fitzgerald L Whittaker M Tanner M The architecture and effect of participation: a systematic review of community participation for communicable disease control and elimination. Implications for malaria elimination Malar J 2011 14 225 21816085 10.1186/1475-2875-10-225 PMC3171376

[B34] Myers KM Palmer NB Geyer JR Research in child and adolescent telemental health Child Adolesc Psychiatr Clin N Am 2011 14 1 155 171 21092919 10.1016/j.chc.2010.08.007

[B35] Roozen HG de Waart R Community reinforcement and family training: an effective option to engage treatment-resistant substance-abusing individuals in treatment [corrected] [published erratum appears in ADDICTION 2010 Nov;105(11):2040] Addiction 2010 14 10 1729 1738 20626372 10.1111/j.1360-0443.2010.03016.x

[B36] Graven C Brock K Hill K Joubert L Are rehabilitation and/or care co-ordination interventions delivered in the community effective in reducing depression, facilitating participation and improving quality of life after stroke? Disabil Rehabil 2011 14 17/18 1501 1520 21204742 10.3109/09638288.2010.542874

[B37] Rabinowitz YG Gallagher-Thompson D Recruitment and retention of ethnic minority elders into clinical research Alzheimer Dis Assoc Disord 2010 14 SUPPL. 1 S35 S41 22720320

[B38] Grill JD Karlawish J Addressing the challenges to successful recruitment and retention in Alzheimer’s disease clinical trials Alzheimers Res Ther 2010 14 6 34 21172069 10.1186/alzrt58 PMC3031880

[B39] Yancey AK Ortega AN Kumanyika SK Effective recruitment and retention of minority research participants Annu Rev Public Health 2006 14 1 28 16533107 10.1146/annurev.publhealth.27.021405.102113

[B40] Dancy BL Wilbur J Talashek M Bonner G Barnes-Boyd C Community-based research: barriers to recruitment of African Americans Nurs Outlook 2004 14 5 234 240 15499312 10.1016/j.outlook.2004.04.012

[B41] Goss C Mosconi P Renzi C Deledda G Participation of patients and citizens in healthcare decisions in Italy ZEFQ 2011 14 4 277 282 10.1016/j.zefq.2011.04.003 21620321

[B42] Cornuz J Kuenzi B Krones T Shared decision making development in Switzerland: room for improvement! ZEFQ 2011 14 4 296 299 10.1016/j.zefq.2011.04.008 21620324

[B43] Dubois JM Bailey-Burch B Bustillos D Campbell J Cottler L Fisher CB Hadley WB Hoop JG Roberts L Salter EK Sieber JE Stevenson RD Ethical issues in mental health research: the case for community engagement Curr Opin Psychiatry 2011 14 3 208 214 21460643 10.1097/YCO.0b013e3283459422 PMC3528105

[B44] Boote J Baird W Beecroft C Public involvement at the design stage of primary health research: a narrative review of case examples Health Policy 2010 14 1 10 23 19963299 10.1016/j.healthpol.2009.11.007

[B45] Boote J Baird W Sutton A Public involvement in the systematic review process in health and social care: a narrative review of case examples Health Policy 2011 14 2–3 105 116 21641075 10.1016/j.healthpol.2011.05.002

[B46] Doughty C Tse S Can consumer-led mental health services be equally effective? an integrative review of CLMH services in high-income countries Community Ment Health J 2011 14 3 252 266 20512528 10.1007/s10597-010-9321-5

[B47] Forbes A While A Griffiths P Ismail K Heller S Organizing and delivering diabetes education and self-care support: findings of scoping project J Health Serv Res Policy 2011 14 Suppl 1 42 49 21460349 10.1258/jhsrp.2010.010102

[B48] Macdonald G Turner W Treatment foster care for improving outcomes in children and young people Cochrane Database Syst Rev 2011 14 5 10.1002/14651858.CD005649.pub2 18254087

[B49] Lassi ZS Haider BA Bhutta ZA Community-based intervention packages for reducing maternal and neonatal morbidity and mortality and improving neonatal outcomes Cochrane Database Syst Rev 2011 14 1 10.1002/14651858.CD007754.pub2 21069697

[B50] Maticka-Tyndale E Barnett JP Peer-led interventions to reduce HIV risk of youth: a review Eval Program Plann 2010 14 2 98 112 19647874 10.1016/j.evalprogplan.2009.07.001

[B51] Wright-Berryman JL McGuire AB Salyers MP A review of consumer-provided services on assertive community treatment and intensive case management teams: implications for future research and practice J Am Psychiatr Nurses Assoc 2011 14 1 37 44 21659293 10.1177/1078390310393283 PMC3117264

[B52] Repper J Carter T A review of the literature on peer support in mental health services J Ment Health 2011 14 4 392 411 21770786 10.3109/09638237.2011.583947

[B53] Curtis-Tyler K Levers and barriers to patient-centred care with children: findings from a synthesis of studies of the experiences of children living with type 1 diabetes or asthma Child Care Health Dev 2011 14 4 540 550 21143267 10.1111/j.1365-2214.2010.01180.x

[B54] Legare F Stacey D Forest PG Coutu MF Moving SDM forward in Canada: milestones, public involvement, and barriers that remain ZEFQ 2011 14 4 245 253 10.1016/j.zefq.2011.04.011 21620316

[B55] Claes C Van Hove G Vandevelde S van Loon J Schalock RL Person-centered planning: analysis of research and effectiveness Intellect Dev Disabil 2010 14 6 432 453 21166549 10.1352/1934-9556-48.6.432

[B56] Bruegel RB Patient empowerment-a trend that matters J AHIMA 1998 14 8 30 10182504

[B57] Samoocha D Bruinvels DJ Elbers NA Anema JR van der Beek AJ Effectiveness of web-based interventions on patient empowerment: a systematic review and meta-analysis J Med Internet Res 2010 14 2 e23 20581001 10.2196/jmir.1286 PMC2956234

[B58] Ennis L Rose D Callard F Denis M Wykes T Rapid progress or lengthy process? electronic personal health records in mental health BMC Psychiatry 2011 14 117 21791069 10.1186/1471-244X-11-117 PMC3163520

[B59] Parsons T The sick role and the role of the physician reconsidered Milbank Mem Fund Q Health Soc 1975 14 3 257 278 1041510

[B60] Evans D Rhetoric or reality? A systematic review of the impact of participatory approaches by UK public health units on health and social outcomes J Public Health 2010 14 3 418 426 10.1093/pubmed/fdq014 20194176

[B61] Sarrami Fouroushani P Travaglia JF Eikli M Braithwaite J Consumer and Community Engagement: A Review of The Literature 2012 Sydney: University of New South Wales, Centre for Clinical Governance Research, Australian Institute of Health Innovation

